# Development of β-Lactoglobulin-Specific Chimeric Human IgEκ Monoclonal Antibodies for *In Vitro* Safety Assessment of Whey Hydrolysates

**DOI:** 10.1371/journal.pone.0106025

**Published:** 2014-08-25

**Authors:** Karen Knipping, Peter J. Simons, Laura S. Buelens-Sleumer, Linda Cox, Marcel den Hartog, Niels de Jong, Reiko Teshima, Johan Garssen, Louis Boon, Léon M. J. Knippels

**Affiliations:** 1 Nutricia Research B.V., Utrecht, The Netherlands; 2 Division of Pharmacology, Utrecht Institute for Pharmaceutical Sciences, Utrecht University, Utrecht, The Netherlands; 3 Bioceros Holding B.V., Utrecht, The Netherlands; 4 Division of Foods, National Institute of Health Sciences, Tokyo, Japan; King's College London, United Kingdom

## Abstract

**Background:**

Cow’s milk-derived whey hydrolysates are nutritional substitutes for allergic infants. Safety or residual allergenicity assessment of these whey hydrolysates is crucial. Currently, rat basophilic leukemia RBL-2H3 cells expressing the human IgE receptor α-chain (huFcεRIα-RBL-2H3), sensitized with serum IgE from cow’s milk allergic children, are being employed to assess *in vitro* residual allergenicity of these whey hydrolysates. However, limited availability and inter-lot variation of these allergic sera impede standardization of whey hydrolysate safety testing in degranulation assays.

**Objective:**

An oligoclonal pool of chimeric human (chu)IgE antibodies against bovine β-lactoglobulin (a major allergen in whey) was generated to increase sensitivity, specificity, and reproducibility of existing degranulation assays.

**Methods:**

Mice were immunized with bovine β-lactoglobulin, and subsequently the variable domains of dissimilar anti-β-lactoglobulin mouse IgG antibodies were cloned and sequenced. Six chimeric antibodies were generated comprising mouse variable domains and human constant IgE/κ domains.

**Results:**

After sensitization with this pool of anti-β-lactoglobulin chuIgEs, huFcεRIα-expressing RBL-2H3 cells demonstrated degranulation upon cross-linking with whey, native 18 kDa β-lactoglobulin, and 5–10 kDa whey hydrolysates, whereas a 3 kDa whey hydrolysate and cow’s milk powder (mainly casein) showed no degranulation. In parallel, allergic serum IgEs were less sensitive. In addition, our pool anti-β-lactoglobulin chuIgEs recognized multiple allergenic immunodominant regions on β-lactoglobulin, which were also recognized by serum IgEs from cow’s milk allergic children.

**Conclusion:**

Usage of our ‘unlimited’ source and well-defined pool of β-lactoglobulin-specific recombinant chuIgEs to sensitize huFcεRIα on RBL-2H3 cells showed to be a relevant and sensitive alternative for serum IgEs from cow’s milk allergic patients to assess safety of whey-based non-allergic hydrolyzed formula.

## Introduction

Food allergens, aeroallergens, medications and insect venoms are the most common allergens which are responsible for inducing Type I or immunoglobulin E (IgE)-mediated hypersensitivity reactions [1]. Type I allergic responses to cow’s milk (CM) proteins leading to cow’s milk allergy (CMA) are characterized by a T helper 2 response resulting in the production of allergen-specific IgEs. Binding of these IgEs to high affinity IgE receptors (FcεRI) on mast cells or basophils, followed by cross-linking of these IgEs by allergens, elicits degranulation and release of mediators, e.g. histamine, leukotrienes, and inflammatory cytokines. The optimal conditions for this release depend on the concentration of membrane-bound allergen-specific IgEs, the concentration of allergen and the affinity of the IgE for the allergen [2], [3]. Clinical symptoms may occur in the skin, gastrointestinal tract, and airways and can even result in life threatening anaphylactic shock.

CMA is the most common allergy in early childhood, with an estimated prevalence of 3% in the pediatric population [4]. While breastfeeding is considered the golden standard for infant nutrition, hypoallergenic (HA) formulas are a good alternative for infants at risk of developing allergy or for infants diagnosed with CMA. HA formulas are processed by enzymatic treatment, heat treatment and/or ultrafiltration of CM proteins. These hydrolyzed formulas are generally categorized as either partial or extensive hydrolysates based on the degree of hydrolysis, and can be characterized by assessing the molecular weight distribution of the residual proteins or peptides. However, residual allergenicity cannot be ascertained solely based on peptide size distribution. Several clinical studies have been performed to address the sensitizing capacity of partial and extensive HA formulas in high risk children [5]–[8]. Differences in peptide size, variations in protein sources and hydrolysis methods can modify the hypoallergenic nature of these formulas [9] stressing the importance of adequate pre-clinical testing. Animal models to assess the allergenicity of HA formulas are developed but never fully validated. However, for safety reasons, the hypoallergenicity of hydrolyzed infant formulas needs to be assessed by showing that these formulas are not able to sensitize animals to the protein source they are derived from (Commission Directive 96/4/EC of 16th February 1996 amending Directive 91/321/EEC on infant formulae and follow-on formulae. Official Journal of the European Communities No L 49: 12–16). Therefore, active systemic anaphylaxis assays in orally sensitized guinea pigs have been commonly used for this purpose. A major disadvantage of this guinea pig model is the generation of anaphylactic IgG1a instead of IgE antibodies [10], which are the main physiological antibody responses in allergic humans. Currently, a CMA mouse model to assess the allergenicity of hydrolyzed CM based infant formulas is being evaluated [11]. Assessment of residual allergenicity of hydrolyzed formulas by peptide size distribution analysis, residual allergen detection by ELISA and SDS-PAGE/western blotting followed by immune incubation with specific antibodies in combination with *in vitro* cellular degranulation assays is proposed as a strategy for the screening of new HA formulas aimed at preventing sensitization in atopic children and avoiding clinical symptoms in infants suffering from CMA [12]. One of these proposed *in vitro* cellular degranulation assays uses the rat basophilic leukaemia cell line RBL-2H3 stably transfected with the α-chain of the human FcεRI (RBL-huFcεRI) as target cells. This α-chain of human FcεRI associates with one β-chain and two γ-chains of rat FcεRI, and forms functional high affinity IgE receptor complexes on the surface of RBL-huFcεRI cells. The usefulness of RBL-huFcεRI cell degranulation after exposure to IgE containing serum from allergic patients, and subsequent cross-linking with either anti-human IgE antibodies [13] or with multiple allergens [14]–[16] has been extensively tested and established by several investigators. Though these studies clearly demonstrated that RBL-huFcεRI cells form the basis of a relevant *in vitro* model system for the detection of allergens and for the study of IgE-allergen interactions [13], [14], it was also demonstrated that serum contained factors that blocked IgE binding to FcεRI or induced cross-linking of membrane-bound IgE by anti-human IgE antibodies [13]. Furthermore, Marchand et al. found a poor correlation between human serum IgE-mediated RBL-huFcεRI cell degranulation and allergen-specific IgE content in serum [15]. Ladics et al. concluded that (i) there was no consistent RBL-huFcεRI cell degranulation with a potent food allergen, and that (ii) the RBL-huFcεRI cell degranulation assay's utility in safety assessment, predictive value and reproducibility for evaluating the cross-reactivity of proteins with allergens needed further investigation with additional proteins and well-characterized sera [16].

Whey proteins represent nearly 20% of the total CM proteins. The known major allergenic proteins of whey are β-lactoglobulin (BLG) and α-lactalbumin (ALA) [17]. To assess the residual allergenicity of whey hydrolysates, currently a pooled serum of at least ten CMA patients is used; however, this has many disadvantages. Onset of CMA is usually in the first year of life and outgrown after the third year of life in about 85% of these infants. Therefore, the amounts of serum available for use in the degranulation assay with RBL-huFcεRI cells are very limited, which makes it difficult to obtain a large, well-validated pool of serum. It has also been demonstrated that even when the CM-specific IgE concentration in an individual serum sample was high, in some of these sera no binding of IgE to the RBL-huFcεRI cells was detected. Another observation was that the spontaneous degranulation of the RBL-huFcεRI with serum alone could become very high, possibly by other intervening or cytotoxic substances present in serum, which considerably lower the sensitivity of the assay [18], [19]. Therefore, there is a need of a well-defined whey-specific IgE antibody pool to standardize the RBL-huFcεRI assay for safety assessment of whey hydrolysates.

In this study, the development of six BLG-specific chimeric human IgE monoclonal antibodies covering allergenic epitopes of BLG is described. The optimal concentration of our pool of BLG-specific chimeric IgE monoclonal antibodies for use in the RBL-huFcεRI degranulation assay was established and tested with whey hydrolysates with different degrees of hydrolysis to assess sensitivity and specificity.

## Methods

### Ethic statement

The Immunization protocol described below was approved (approval no. 2009.III.03.023) by the Animal Experiments Committee of the Faculty of Veterinary Medicine, Utrecht University, The Netherlands.

The sera used for epitope mapping was from a cohort study that was performed in children successively referred with eosinophilic esophagitis to the pediatric GI department of Hospital Necker-Enfants malades, Paris, France. Participants were recruited from the ARSENE cohort, a collection of blood samples registered within the French Ministry of Health (number DC-2009-955) [20]. According to French law, such a collection allowing to keep routinely collected material needs a declaration to the French ministry of Health and a written informed consent obtained from all parents and a written informed consent from children 11 years and older. In contrast, no involvement of Ethics committees is requested. Blood samples were collected by venipuncture and serum samples are stored in the Biological Resource Platform (Necker Biobank) of the Hospital Necker-Enfants malades. All data has been anonymized.

### Immunization and generation of antibodies against whey

BALB/c mice (females, 6 weeks of age; Charles River Laboratories) were subcutaneously injected with bovine whey (Lacprodan WPC80, Arla Foods), each mouse received 200 µg bovine whey in 200 µl PBS mixed with 200 µl Complete Freund’s adjuvant (Sigma) at day 0. Antibody responses in mice were then boosted by subcutaneous injections of bovine whey, each mouse received 200 µg bovine whey in 200 µl PBS mixed with 200 µl Incomplete Freund’s adjuvant (Sigma) at day 21 and day 42. This was followed by intraperitoneal injections with bovine whey without adjuvant, each mouse received 200 µg bovine whey in 200 µL PBS at day 61 and day 62. At day 65, splenocytes from immunized mice were fused with SP2/0-Ag14 myeloma cells (DSMZ) using slightly modified hybridoma technology originally described by Köhler and Milstein [21]. Briefly, splenocytes were teased from spleens and washed in serum-free opti-MEM I with GlutaMax medium (SF medium; Invitrogen). Logarithmically growing SP2/0-Ag14 myeloma cells were washed in SF medium and added to the splenocytes yielding a 5∶1 ratio of splenocytes-to-myeloma cells. Cells were then pelleted and supernatant was removed. One ml of a 37% (v/v) solution of polyethylene glycol 4000 (Merck) was then added dropwise over a 60 second period, after which the cells were incubated for another 60 seconds at 37°C. Eight ml SF medium, followed by 5 ml opti-MEM I with GlutaMax/10% (v/v) fetal calf serum (FCS; Bodinco), was then slowly added with gentle agitation. After incubation of 30 minutes at room temperature (RT), cells were pelleted, washed in opti-MEM I with GlutaMax/10% FCS to remove residual polyethylene glycol, and finally plated at a concentration of 10^5^ cells/200 µl per well in aminopterin selection medium, i.e., opti-MEM I with GlutaMax/10% FCS that was supplemented with 50x Hybri-Max aminopterin (a de novo DNA synthesis inhibitor (Sigma)). From day 7 after fusion, aminopterin selection medium was replenished every 2–3 days, and at day 16, aminopterin selection medium was replaced by opti-MEM I with GlutaMax/10% FCS.

From day 16 after fusion, supernatants from hybridomas were screened for anti-whey IgG antibody production using a standard ELISA with bovine whey (Lacprodan WPC80). Briefly, bovine whey was coated (100 ng whey/100 µl PBS/well) onto EIA plates (Corning) o/n at 4°C. After washing with PBS/0.05% Tween 20, plates were blocked with PBS/0.05% Tween 20/10% ELISA Blocking Reagent (Roche) for 1 hour at RT. Subsequently, plates were incubated with 100 µl serially-diluted hybridoma supernatant per well for 1 hour at RT. After washing in PBS/0.05% Tween 20, binding of IgG antibodies was determined with 1∶5,000 diluted HRP-conjugated goat anti-mouse IgG-specific antibodies (Jackson ImmunoResearch) for 1 hour at RT, followed by a ready-to-use solution of TMB substrate (Invitrogen) for colorimetric detection. After adding 1 M H_2_SO_4_, optical densities were measured at a wavelength of 450 nm (reference wavelength of 655 nm) using a microplate reader (BioRad). Hybridomas positive in this whey ELISA were expanded and cryopreserved.

Fusions yielded 40 hybridomas with high IgG antibody titers against bovine whey. Next, supernatants of these anti-whey producing hybridomas were tested for their fine-specificity against bovine BLG and bovine ALA, the main constituents of bovine whey. Briefly, whey-purified BLG or ALA (both Davisco Foods International) was coated (10 and 50 ng/100 µl PBS/well) onto EIA plates (Corning) o/n at 4°C. After incubation, 10-fold diluted supernatants of the 40 anti-whey producing hybridomas were examined using the same whey ELISA method as described above. This screening resulted in 15 hybridomas that produced IgG antibodies specifically against bovine BLG, 7 hybridomas that produced IgG antibodies against bovine ALA, and 18 hybridomas against non-BLG/non-ALA proteins (most likely against whey-derived bovine IgG and bovine serum albumin).

### Determination of (non)cross-blocking of mouse anti-BLG antibodies using a competitive BLG ELISA test

Recognition of multiple dissimilar epitopes on BLG by BLG-specific IgE antibodies on sensitized FcεRI expressing RBL cells is a prerequisite for successful IgE/BLG cross-linking, and subsequent degranulation. In order to identify mouse anti-BLG IgG antibodies, which recognized similar or dissimilar parts on BLG, a competitive BLG ELISA test was performed. For this, a selection protein G-purified mouse anti-BLG IgG antibodies from hybridomas with highest antibody titers designated as 7D5, 8C3, 8F7, and 13A5, was labeled with *N*-hydroxysuccinimido-biotin (Pierce). Then, whey-purified bovine BLG was coated (200 ng/100 µl PBS/well) onto EIA plates (Corning) o/n at 4°C. After blocking with PBS/0.05% Tween 20/1% BSA fraction V (Roche), BLG was saturated with each individual unlabeled mouse anti-BLG IgG antibodies 7D5, 8C3, 8F7, or 13A5 (2.5 µg/100 µl PBS/well) for 1 hour at RT, followed by an incubation with suboptimal concentrations of each individual biotinylated mouse anti-BLG antibodies 7D5, 8C3, 8F7, or 13A5 for 1 hour RT. Binding of biotinylated antibodies was determined with 1∶5,000 diluted HRP-conjugated streptavidin (Jackson ImmunoResearch) for 1 hour at RT, followed by a ready-to-use solution of TMB substrate (Invitrogen) for colorimetric detection.

### Generation of chimeric mouse/human IgE and IgG1 anti-BLG antibodies

After selection of the lead hybridomas that produced mouse (non)cross-blocking anti-BLG IgG antibodies (all lead mouse antibodies were of the IgG1κ isotype), these hybridomas were subcloned (by limiting dilution), and designated as clone 5D6.1, 7D5.1, 8C3.3, 8F7.1, 11B6.2, and 13A5.2. Then, 5×10^6^ viable cells from each subcloned hybridoma were washed in PBS and frozen at −80°C. From these cell pellets, total RNA was isolated and purified using an RNeasy mini kit (Qiagen) according to manufacturer's instructions. Purified RNA (1–2 µg) was then used as templates for cDNA synthesis with a RevertAid H Minus First Strand cDNA synthesis kit (Fermentas). Next, variable light (VL) and variable heavy (VH) regions were amplified in PCR reactions comprising 25 ng cDNA, 10x PCR mix containing dNTPs (Life Technologies), 25 pmol 5′ and 3′ primers (Biolegio) described in Table 1, and AccuPrimeTM Pfx DNA Polymerase (Life Technologies) according to manufacturer’s instructions. Amplification consisted of initialization at 95°C for 2 minutes, 13 cycles of denaturation at 95°C for 30 seconds, annealing at gradient 42–55°C for 30 seconds, and extension at 68°C for 2.5 minutes, followed by 23 cycles under identical conditions, however, with annealing at 55°C and extension for 7 minutes. Gel-purified PCR products were cloned into the pCRII blunt TOPO vector (Life Technologies) and transformed into DH5α E.coli. Plasmid DNA was isolated from several clones using a QIAprep spin miniprep kit (Qiagen) according to manufacturer's instructions. Subsequently, consensus DNA sequences of VL and VH regions from each monoclonal mouse anti-BLG antibody were determined by DNA sequencing (ServiceXS B.V., Leiden, The Netherlands) of several plasmid clones. Based on these consensus V-region sequences, cDNAs encoding chimeric mouse/human IgEκ and IgG1κ anti-BLG antibodies were designed. For anti-BLG antibody clones 5D6.1, 7D5.1, 8C3.3, 8F7.1, 11B6.2, and 13A5.2, cDNAs encoding mouse VH regions linked to a human IgE constant heavy chain (CH1–CH2–CH3–CH4; GenBank accession no. P01854.1), and for anti-BLG antibody clones 7D5.1, 8F7.1, and 13A5.2, cDNAs encoding either mouse VL regions linked to a human κ constant light chain (GenBank accession no. P01834.1) or mouse VH region linked to a human IgG1 constant heavy chain (CH1–CH2–CH3; GenBank accession no. P01857.1) were ordered at Life Technologies/GENEART. These cDNAs were recloned with HindIII/XhoI from the GENEART shuttle vector in expression plasmids, i.e., pcDNA3.1 containing a longer CMV promoter combined with either zeocin (chimeric κ chains) or blasticidin (chimeric IgE and IgG1 chains) as selection markers. For anti-BLG antibody clones 8C3.3, 5D6.1, and 11B6.2, cDNA gene constructs encoding either chimeric κ light chains or chimeric IgG1 heavy chains were generated by swapping V-regions in chimeric κ or IgG1 chains of anti-BLG antibody clone 13A5.2 expression plasmids with respective VL and VH from the former 3 anti-BLG antibodies. By using 5′ and 3′ primers annealing to VL and VH regions of anti-BLG antibody clones 8C3.3, 5D6.1, and 11B6.2, respectively NheI/SacII and RsrII/SacII restriction sites were introduced in the V-regions by PCR. PCR was done following standard procedures with AccuPrimeTM Pfx DNA Polymerase as described above without the gradient step. Amplified PCR products were gel purified, and subcloned in a pCRII blunt TOPO vector (Life Technologies). cDNA sequences were confirmed by sequencing. For each antibody using NheI/SacII or RsrII/SacII restriction sites, V-regions were swapped. For each antibody, finally 3 expression plasmids were obtained: one containing cDNA encoding the chimeric κ light chain, one encoding the chimeric IgG1 heavy chain, and one encoding the chimeric IgE heavy chain. From all 18 expression vectors, DNA was prepared using an Endofree plasmid maxi kit (Qiagen). Finally, DNA preparations were checked by restriction enzyme analysis, and DNA inserts with genes of interest were sequenced.

**Table 1:** Overview of PCR primer sets for each antibody to amplify VL and VH regions.

antibody	region	5′ primer	3′ primer
8F7.1	VL	GCGCTTAACACAAACNCCNYT	GACAGTTGGTGCAGCATCAG
	VL	GCGCGACGTAGTAYTNACNCARWSNCC	GACAGTTGGTGCAGCATCAG
	VH	GCGCGAAATTCAAYTNCARCARACNGG	GGCCAGTGGATAGACAGATG
	VH	GCGCGAAATTCAAYTNCARCARACNGG	TGGACAGGGATCCAGAGTTC
5D6.1	VL	GCGCTTAACACAAACNCCNYT	ACACTCATTCCTGTTGAAGCTCTTG
	VH	GCGCGAAATTCAAYTNCARCARACNGG	AATTTTCTTGTCCACYTTGGTGCT
11B6.2	VL	GCGATATACARATGACNCARAC	ACACTCATTCCTGTTGAAGCTCTTG
	VH	SAGGTSMARCTGVAGSAGTCWGG	AATTTTCTTGTCCACYTTGGTGCT
7D5.1	VL	GCGATATACARATGACNCARAC	ACACTCATTCCTGTTGAAGCTCTTG
	VH	SAGGTSMARCTGVAGSAGTCWGG	AATTTTCTTGTCCACYTTGGTGCT
13A5.2	VL	GCGATATACARATGACNCARAC	ACACTCATTCCTGTTGAAGCTCTTG
	VH	SAGGTSMARCTGVAGSAGTCWGG	AATTTTCTTGTCCACYTTGGTGCT
8C3.3	VL	GACAGTTGGTGCAGCATCAG	GACAGTTGGTGCAGCATCAG
	VH	SAGGTSMARCTGVAGSAGTCWGG	AATTTTCTTGTCCACYTTGGTGCT

The sense VH and VL primers (5′ primers) anneal to framework 1 sequences of murine antibodies based on GENBANK searches; the antisense VH and VL primers (3′ primers) anneal to the constant regions of murine IgG1 and murine kappa chain, respectively.

Chimeric IgEκ or IgG1κ (IgG1κ class was also produced for blocking purposes; see Results section) anti-BLG antibodies were generated by combining expression plasmids containing cDNA encoding chimeric κ light chains with their corresponding plasmids encoding chimeric IgE heavy chains or chimeric IgG1 heavy chains. To this end, 300 ml CHO-S cells (Life Technologies) were transiently transfected using these plasmids (at a heavy-to-light chain plasmid ratio of 1∶1 (w/w); plasmids have similar sizes) with FreeStyle MAX reagent (Life Technologies). Cultures were incubated for several days at 36.5°C and 5% CO_2_ under humidified conditions on an orbital shaker at 110 rpm. Supernatants were harvested for antibody purification when cellular viability dropped below 50%.

### Purification of chimeric mouse/human IgE and IgG1 anti-BLG antibodies

Supernatants from transiently transfected CHO cells were collected, and then cleared by centrifugation at 3500 g for 10 minutes at RT. Chimeric anti-BLG antibodies were purified using either HiTrap KappaSelect (chimeric human IgE antibodies) or HiTrap protein A HP (chimeric human IgG1 antibodies) columns (GE Healthcare) on an ÄKTAprime plus liquid chromatography system (GE Healthcare). Briefly, culture supernatants were adjusted to pH 7.2 and loaded onto affinity columns. After washing with PBS pH 7.2, chimeric anti-BLG antibodies were eluted from affinity columns with 0.1 M glycine buffer pH 3.0, and instantly neutralized in 1 M Tris buffer pH 9.0. After dialysis against PBS pH 7.2, antibody concentrations were determined by measuring the absorbance at 280 nm. Extinction coefficients (1 mg/ml at 1 cm pathway) of 1.53 and 1.36 [22] were used for chimeric IgE antibodies and chimeric IgG1 antibodies, respectively. Purity of chimeric anti-BLG antibodies was analyzed with SDS-PAGE using a pre-casted 4–12% BisTris gel/MOPS running buffer NuPage Novex system (Invitrogen) followed by Coomassie Brilliant Blue staining (BioRad). MW Magic ladder (BioRad) was run in parallel to estimate the molecular weight of proteins.

### Binding of purified chimeric mouse/human IgE and IgG1 anti-BLG antibodies against bovine BLG using ELISA and Western blotting

Binding of purified chimeric human IgE and IgG1 anti-BLG antibodies clones 5D6.1, 7D5.1, 8C3.3, 8F7.1, 11B6.2 or 13A5.2 was done by using the BLG ELISA as described above. For detection, however, 1∶1,000 diluted HRP-conjugated goat anti-human IgE-specific antibodies (Sigma) and 1∶5,000 diluted HRP-conjugated goat anti-human IgG-specific antibodies (Jackson ImmunoResearch) were applied.

Bovine BLG (120 µg/480 µl non-reducing sample buffer) was loaded and electrophoresed in a one well pre-casted 4–12% BisTris gel/MOPS running buffer NuPage Novex system (Invitrogen). Then, bovine BLG was electro-blotted onto a polyvinylidene fluoride transfer membrane (Millipore). After blocking with PBS/0.05% Tween 20/1% BSA fraction V (Roche) for 1 hour at RT, this membrane was incubated with 1 µg/ml purified chimeric human IgE anti-BLG antibody clones 5D6.1, 7D5.1, 8C3.3, 8F7.1, 11B6.2 or 13A5.2 for 1 hour at RT. After washing in PBS/0.05% Tween 20, binding of these chimeric human IgE anti-BLG antibodies was determined with 1∶4,000 diluted HRP-conjugated goat anti-human IgE-specific antibodies (Sigma) for 1 hour at RT, followed by a ready-to-use solution of TMB substrate (Sigma) for colorimetric detection.

### Binding of purified chimeric mouse/human IgE anti-BLG antibodies against human FcεRI expressing on RBL cells using flow cytometry

RBL-huFcεRI cells (see below) were adjusted to 1×10^6^ cells/ml in ice-chilled PBS/0.1% BSA (Sigma)/0.01% NaN_3_ (Acros). Subsequently, RBL cells were incubated with 20 µg/ml purified chimeric human IgE anti-BLG antibody clones 5D6.1, 7D5.1, 8C3.3, 8F7.1, 11B6.2 or 13A5.2 for 30 minutes at 4°C. After washing in ice-chilled PBS/0.1% BSA/0.01% NaN_3_, binding of chimeric huIgE anti-BLG antibodies was detected using 1∶10 diluted FITC-labeled goat anti-human IgE-specific antibodies (KPL) for 30 minutes at 4°C. For human FcεRI expression levels, RBL cells were incubated with a 1∶5 diluted PE-labeled mouse anti-human FcεRI α chain-specific antibody (eBioscience) for 30 minutes at 4°C. RBL cells were fixed in PBS/0.1% BSA/0.01% NaN_3_/2% formaldehyde (Sigma) for 30 minutes at 4°C prior to flow cytometric measurements using FACSCalibur (BD Biosciences).

### Degranulation of RBL-huFcεRI cells (RBL-hεIa-2B12 cells)

The cell-line RBL-hεIa-2B12, which was transfected with the α-chain of human Fcε receptor type 1 (hu) FcεRI complex [13], was used for the RBL-huFcεRI assay. Degranulation of RBL-huFcεRI cells with pooled serum from CMA patients was performed as described previously [23]. Confluent growing RBL-huFcεRI cells (1×10^5^/well) in 96-wells flat bottom culture plate were sensitized o/n with 5 µg/ml commercially available human purified IgE (Chemicon/Millipore) and stimulated with 10 µg/ml rabbit anti-human IgE antibodies (Dako) in Tyrode’s buffer at pH 7.4 with 0.1% HSA (Sigma-Aldrich, St. Louis, USA) for 1 hour. This release served as maximum release (100% degranulation). The sensitized cells with single BLG-specific chimeric IgE monoclonal antibodies or a pool of BLG-specific chimeric IgE monoclonal antibodies were stimulated with anti-human IgE (10 µg/ml), milk powder (Friesland Campina; 1 µg/ml in Tyrode’s buffer/HSA), whey (Lacprodan WPC80, Arla Foods; 1 µg/ml in Tyrode’s buffer/HSA), BLG (Davisco Foods International; 1 µg/ml in Tyrode’s buffer/HSA) or whey hydrolysates (Nutricia; experimental hydrolysates <10 kD, <5 kD and <3 kD filtrated over respectively a 10 kD, 5 kD or 3 kD filter; 1 µg/ml in Tyrode’s buffer/HSA) for 1 hour. Therapeutic anti-IgE humanized (huIgG1κ) antibody omalizumab (Xolair; Novartis, Basel, Switzerland), which is well-known for preventing the binding of huIgE on huFcεRI, was applied (at an omalizumab-to-chimeric human IgE molar ratio of 2∶1, 1∶2, 1∶5, and 1∶15) during o/n sensitization of RBL-huFcεRI cells to determine the specificity of the pool of BLG-specific chimeric IgE monoclonal antibodies on degranulation using cross-linking with BLG (1 µg/ml). β-Hexosaminidase activity was determined by a fluorescence assay using 4-methylumbelliferyl-N-acetyl-α-D-glucosamine (Sigma-Aldrich) as a substrate [24]. The β-hexosaminidase activity released into the medium was expressed as the percentage of maximum release observed after cross-linking with anti-human IgE antibodies (set as 100% degranulation, see above).

### Epitope mapping of binding site of BLG-specific chimeric IgE monoclonal antibodies and CMA serum with synthetic BLG peptides

Bovine BLG exists in a range of naturally occurring genetic variants that differ from each other by a few amino acids (AA) substitutions. Two predominant genetic variants of BLG protein are known, A and B [25]. Epitope mapping was done with 25 synthetic peptides (JPT Peptide Technologies GmbH, Berlin, Germany) spanning the BLG of the B whey protein variant and 6 peptides of the A whey protein variant. The peptides are of 18 amino acids (AA) length with 12 AA overlap. Allergic serum samples from 10 patients with high (>50 kU/L) CM-IgE against BLG was a kind gift of Professor Dupont (University Paris-Descartes, Hospital Necker, Paris, France). For epitope mapping an ELISA was performed. In short, wells were coated with 1 µg/well of peptide or of BLG for 16 hours at 4°C. After each incubation step, the wells were washed 3x with 300 µl/well PBS-+0.05% Tween-20 (PBS-T). Subsequently, wells were blocked by incubating with 2% HSA in PBS-T for 2 hours at RT. Wells were incubated for 1 hour at RT with the BLG-specific chimeric IgE monoclonal antibodies (1 µg/ml) or individual sera (1∶20) in 0.5% HSA in PBS-T. Thereafter, wells were incubated for 1 hour with goat anti-human IgE HRP (KPL, Maryland, USA) in 0.5% HSA in PBS-T. TMB substrate solution (Thermo Scientific Pierce, Waltham, USA) was added and incubated for 15 minutes, the reaction was stopped by adding 5 M H_2_SO_4_ (Merck, Whitehouse Station, USA) and absorbance was read at 450 nm. Values of 2x background OD were considered positive. Peptides recognized by 3 or more CMA patient’s sera were considered as relevant allergenic epitopes.

### Statistical analysis

Comparisons were made with (two-tailed and paired) the Student’s *t*-test. *P*-values of less than 0.05 were considered statistically significant.

## Results

### Binding characteristics of lead (non)cross-blocking mouse anti-BLG antibodies

**Figure 1:**
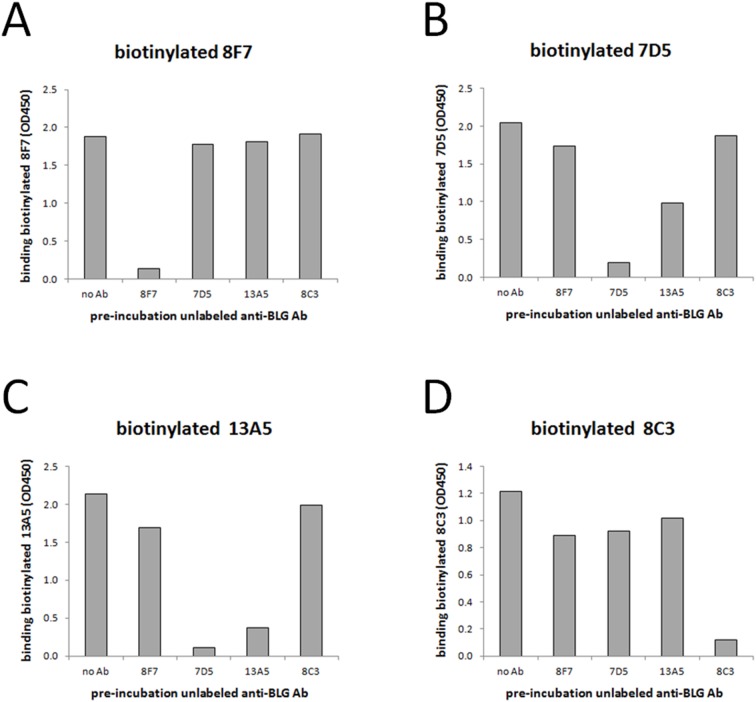
Determination of (non)cross-blocking lead mouse anti-BLG antibodies. BLG-coated ELISA plates were pre-incubated with saturating unlabelled mouse anti-BLG antibodies 8F7, 7D5, 13A5, or 8C3. Subsequently, these plates were incubated with suboptimal concentrations of biotin-labelled 8F7 (at 0.08 µg/mL; A), 7D5 (at 0.08 µg/mL; B), 13A5 (at 0.08 µg/mL; C), or 8C3 (at 0.4 µg/mL; D). Finally, residual binding of these biotinylated mouse anti-BLG antibodies was measured at OD A450 nm. Results (n = 1) are representative of 3–4 independent experiments.

IgE molecules bind to high affinity FcεRIs present on mast cells and basophils, and, through the interaction with a multivalent allergen, trigger release of vasoactive mediators. This process is only feasible when allergen-specific IgE antibodies bind to dissimilar epitopes on a monomeric allergen. However, even when this is the case, allergen-specific IgE antibodies against dissimilar epitopes could prevent their mutual binding to an allergen by sterical hindrance. Therefore, we aimed to generate a panel of mouse anti-BLG antibodies, which did not recognize identical epitopes on BLG and/or did not show any cross-blocking or overlapping occurrence. To this end, we performed a competitive BLG ELISA test. As shown in Figure 1, unlabeled mouse anti-BLG IgG antibodies 7D5, 8C3, 8F7, and 13A5 were able to prevent (≈85–95%) binding of their matching biotinylated antibody counterparts to BLG, which indicated that pre-incubation with unlabeled mouse anti-BLG antibodies was indeed fully saturating BLG. By using this approach, biotinylated anti-BLG antibody 8F7 was still able to bind simultaneously on BLG after pre-incubation with saturating unlabeled anti-BLG antibodies 7D5, 13A5, and 8C3 (Figure 1A), and similarly, biotinylated anti-BLG antibody 8C3 was able to bind simultaneously on BLG after pre-incubation with saturating unlabeled anti-BLG antibodies 8F7, 7D5, and 13A5 (Figure 1D). In contrast, unlabeled anti-BLG antibodies 7D5 and 13A5 seemed to sterically hinder their mutual binding (Figures 1B and 1C), while leaving the binding of unlabeled anti-BLG antibodies 8F7 and 8C3 untouched. Collectively, and for clarity (Table 2), (i) mouse anti-BLG antibody 8F7 was designated as recognizing (arbitrary) domain I on BLG, (ii) cross-blocking (but not necessarily recognizing identical epitopes) mouse anti-BLG antibodies 7D5 and 13A5 were designated as recognizing domain II on BLG, and finally, (iii) mouse anti-BLG antibody 8C3 was designated as recognizing domain III on BLG. This list of lead mouse anti-BLG antibody candidates was extended with mouse anti-BLG antibodies 5D6 and 11B6, which were also designated as recognizing domain I (but not necessarily recognizing identical epitopes), because pre-incubation with saturating chimeric human IgG1 anti-BLG antibody 8F7.1 (see below, *Generation of chimeric human/mouse IgE and IgG1 anti-BLG antibody 8F7.1: proof of principle*) was able to prevent the binding of mouse anti-BLG antibodies 5D6 and 11B6 on BLG (data not shown).

**Table 2:** List of lead (non)cross-blocking mouse anti-BLG antibody candidates for chimerization with human IgEκ and human IgG1κ constant backbones.

Arbitrary domain	Mouse anti-BLG antibody no.
I	8F7, 5D6*, 11B6*
II	7D5, 13A5
III	8C3

Mouse anti-BLG antibodies were examined in a competitive BLG ELISA to determine their (non)cross-blocking binding. *Binding of mouse anti-BLG antibodies 5D6 and 11B6 against BLG was prevented after pre-incubation with saturating chimeric human IgG1 anti-BLG antibody 8F7.1, and therefore designated as recognizing domain I.

### Generation of chimeric mouse/human Ige and Igg1 anti-BLG antibody clone 8F7.1: proof of principle

**Figure 2:**
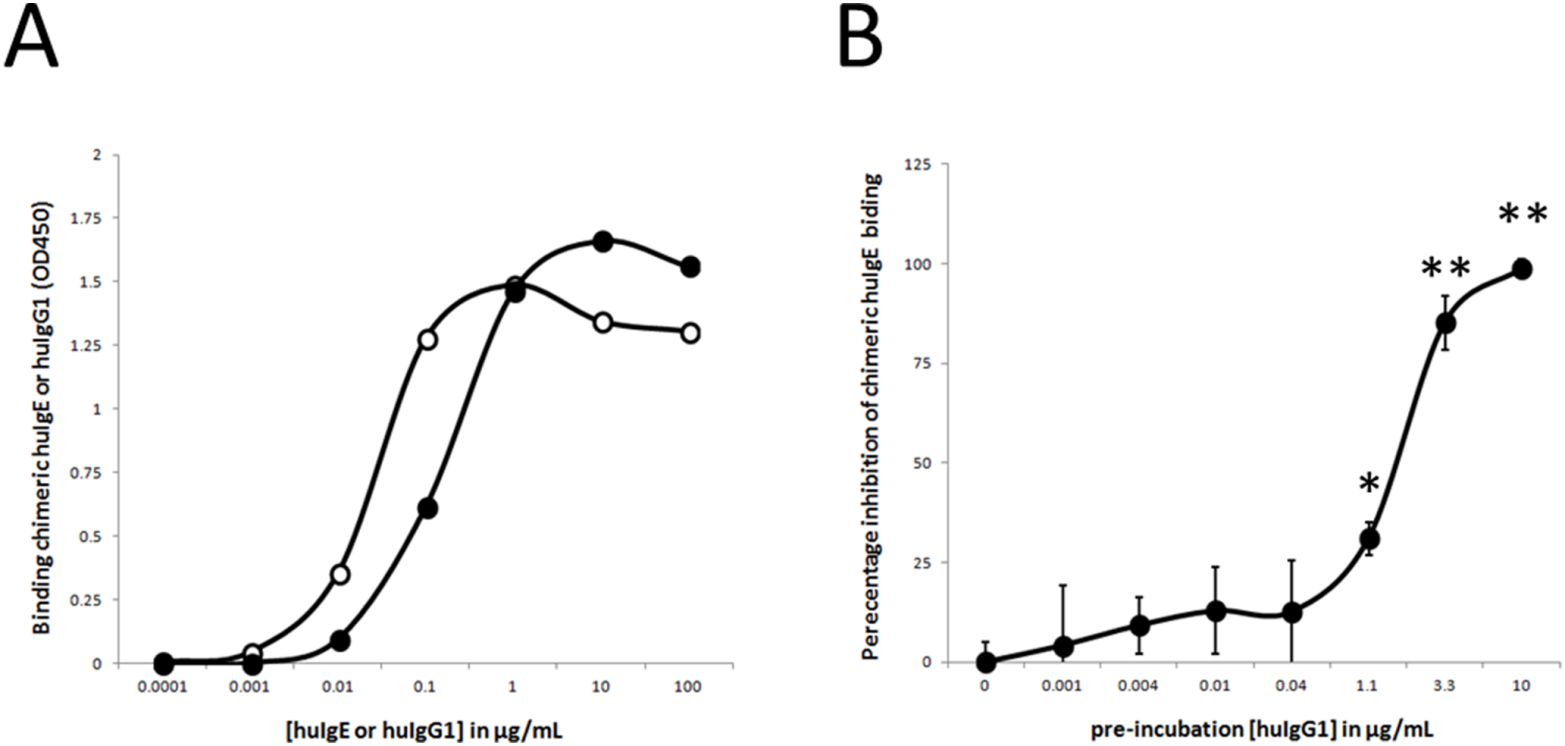
Binding characteristics of lead chimeric huIgE and chimeric huIgG1 anti-BLG antibody clone 8F7.1. (A) BLG-coated ELISA plates were incubated with indicated concentrations of chimeric huIgE (closed symbols) or chimeric huIgG1 (open symbols) anti-BLG antibody clone 8F7.1. Binding of these chimeric anti-BLG antibodies was measured at OD A450 nm. Results (n = 1) are representative of 2 independent experiments. (B) In addition, BLG-coated plates were pre-incubated with indicated concentrations of chimeric huIgG1 anti-BLG antibody clone 8F7.1 followed by adding saturating chimeric huIgE anti-BLG antibody clone 8F7.1. Binding of chimeric huIgE anti-BLG antibody clone 8F7.1 was measured using HRP-labelled huIgE-specific antibodies. Percentage of inhibition of this chimeric huIgE antibody clone 8F7.1 binding by pre-incubated chimeric huIgG1 antibody clone 8F7.1 is shown. Results are expressed as mean ± SD (n = 4); *P<0.01 and **P<0.001 when compared with binding of chimeric IgE anti-BLG antibody clone 8F7.1 without chimeric huIgG1 anti-BLG antibody clone 8F7.1 pre-incubation.

Hybridoma 8F7 was used to provide proof of principle of our chimerization strategy. For this, hybridoma 8F7 cells were subcloned by limiting dilution, which resulted in several hybridoma clones with stable production levels of mouse anti-BLG IgG antibodies. From these stable hybridoma clones, clone 8F7.1 was selected for cloning and sequencing of the VH and VL regions. After chimerization, the binding capacity of purified chimeric human IgE and human IgG1 antibody clone 8F7.1 against BLG was examined. As shown in Figure 2A, both chimeric human antibody classes of clone 8F7.1 bound to BLG in a dose-dependent fashion. Moreover, no binding of both chimeric human antibody classes of clone 8F7.1 was found when BLG coating was omitted or BSA fraction V (at 100 ng/100 µl PBS/well) coating was used (data not shown). Furthermore, pre-incubation with blocking chimeric human IgG1 antibody clone 8F7.1 completely prevented the binding of chimeric human IgE antibody clone 8F7.1 to BLG (Figure 2B), which indicated that (i) both chimeric human antibody classes competed for the same epitope on BLG, and (ii) our chimerization process was successful.

### Generation of chimeric mouse/human Ige and Igg1 anti-BLG antibody clones 5D6.1, 7D5.1 8C3.3, 11B6.2, 13A5.1

**Figure 3:**
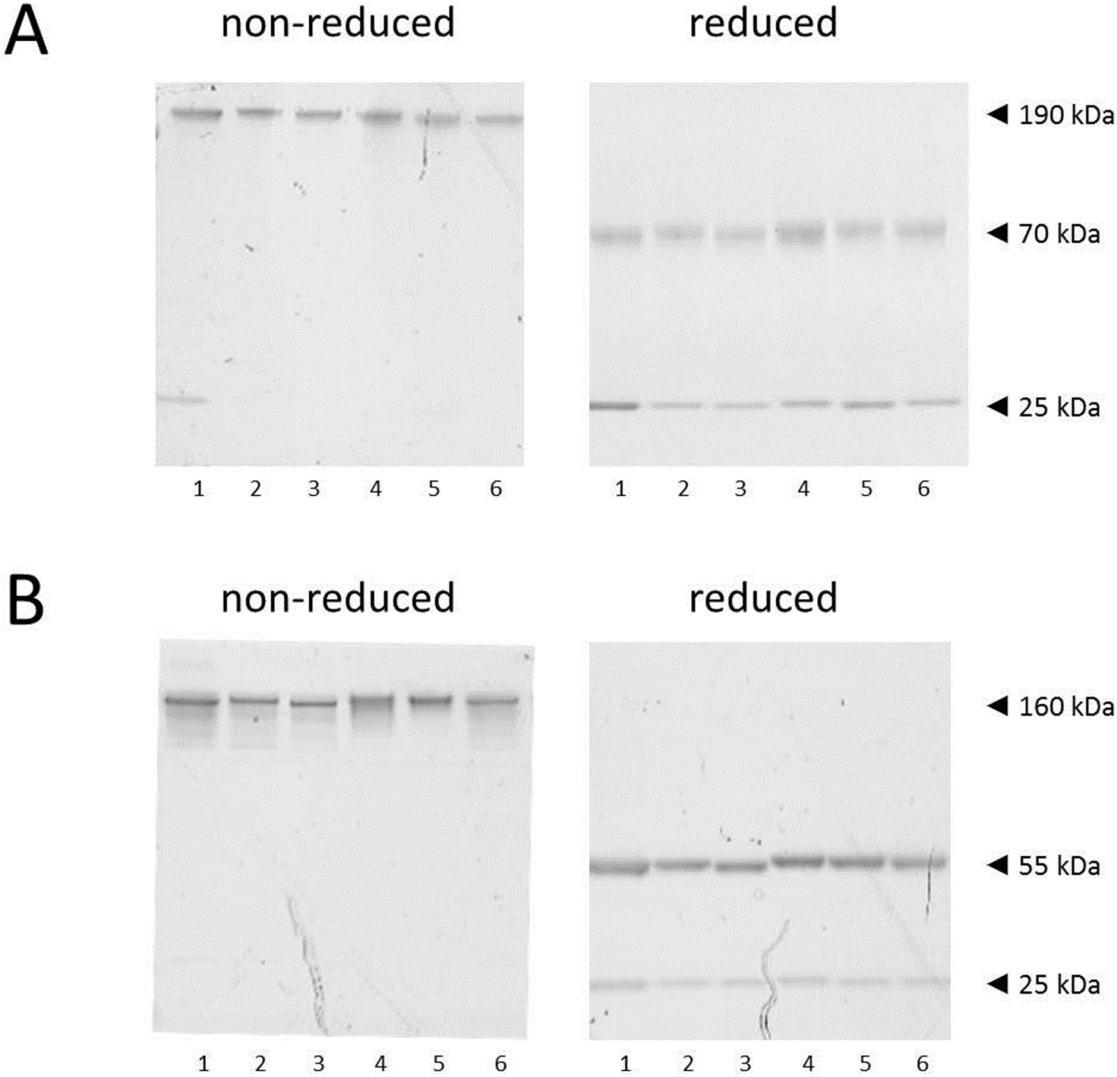
SDS-PAGE analysis of lead chimeric huIgE and chimeric huIgG1 anti-BLG antibody clones 5D6.1, 7D5.1, 8C3.3, 8F7.1, 11B6.2, and 13A5.2. Chimeric huIgE (A) and huIgG1 (B) anti-BLG antibody clones 8F7.1 (lane 1), 5D6.1 (lane 2), 11B6.2 (lane 3), 13A5.2 (lane 4), 7D5.1 (lane 5), and 8C3.3 (lane 6) were loaded onto a 4–12% BisTris gel, and, after electrophoretic separation, proteins were visualized with Coomassie Brilliant Blue. Molecular masses of intact chimeric human IgE and human IgG1 anti-BLG antibodies (non-reduced conditions), and their corresponding heavy and light chains (reduced conditions), are indicated with arrowheads.

Hybridomas 5D6, 7D5, 8C3, 11B6 and 13A5 were also subcloned by limiting dilution. Then, VH and VL regions from stable clones 5D6.1, 7D5.1, 8C3.3, 11B6.2 and 13A5.2 were determined. After chimerization, purity of these chimeric human anti-BLG IgE antibodies was examined as was their binding to bovine BLG (through mouse variable regions of chimeric antibodies) and to human FcεRI α-chain expressing RBL cells (through human Fcε heavy chain tail of chimeric huIgE antibodies). All chimeric human IgE (Figure 3A) and human IgG1 (Figure 3B) anti-BLG antibody preparations were homogenous for >95% using SDS/PAGE. Under non-reducing conditions, predominantly ≈190 kDa and ≈160 kDa proteins were observed in chimeric human IgE and human IgG1 antibody preparations, respectively. After reduction, chimeric κ light chains of ≈25 kDa were observed for both chimeric anti-BLG antibody versions, while chimeric heavy chains consisted of ≈70 kDa and of ≈55 kDa proteins in chimeric human IgE and human IgG1 anti-BLG antibody preparations, respectively. Heavy chains from chimeric human IgE anti-BLG antibodies seemed to be more heavily glycosylated than heavy chains from chimeric human IgG1 anti-BLG antibodies as illustrated by the fuzzy appearance of the former.

**Figure 4:**
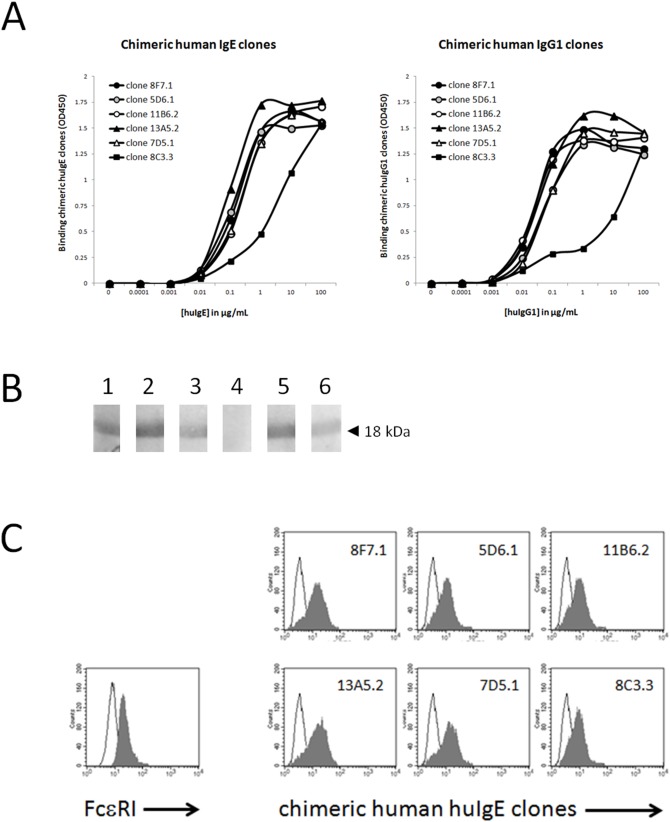
Binding characteristics of lead chimeric huIgE and chimeric huIgG1 anti-BLG antibody clones 5D6.1, 7D5.1, 8C3.3, 8F7.1, 11B6.2, and 13A5.2. (A) BLG-coated ELISA plates were incubated with indicated concentrations of chimeric huIgE or chimeric huIgG1 anti-BLG antibody clones 8F7.1, 5D6.1, 11B6.2, 13A5.2, 7D5.1, and 8C3.3. Binding of these chimeric anti-BLG antibodies was measured at OD A450 nm. Results (n = 1) are representative of 2 independent experiments. (B) Western blotting of non-reduced and denatured bovine BLG (18 kDa), and subsequent detection with chimeric huIgE anti-BLG antibody clones 5D6.1 (lane 1), 7D5.1 (lane 2), 8F7.1 (lane 3), 8C3.3 (lane 4), 11B6.2 (lane 5), and 13A5.2 (lane 6). (C) Human FcεRI α chain expressing RBL cells were incubated with saturating chimeric huIgE anti-BLG antibody clones 8F7.1, 5D6.1, 11B6.2, 13A5.2, 7D5.1, and 8C3.3. Binding of these chimeric huIgE anti-BLG antibodies was flow cytometrically measured after detection with FITC-labelled goat anti-human IgE-specific antibodies. Expression level of human FcεRI α chains on RBL cells was examined with a PE-labelled mouse anti-human FcεRI α chain-specific antibody. White histograms represent background staining and grey histograms represent human FcεRI α chain expression or chimeric human IgE anti-BLG antibody binding on RBL cells.

Next, the binding of our purified chimeric human anti-BLG IgE antibodies against BLG was investigated. As shown in Figure 4A, chimeric human IgE and human IgG1 anti-BLG antibody clones 5D6.1, 7D5.1, 8C3.3, 11B6.2 and 13A5.2 dose-dependently bound to BLG. All chimeric human anti-BLG antibody clones demonstrated similar binding curves with exception of chimeric human - both the IgE and IgG1 version - anti-BLG antibody clone 8C3.3, which resulted in lower OD signals suggestive for a lower binding affinity like observed with its unlabeled (data not shown) and biotinylated (Figure 1; comparison of ODs in D with A-C) parental mouse IgG1 antibody 8C3 versions. Moreover, chimeric human IgE anti-BLG antibody clones 5D6.1, 7D5.1, 8F7.1, 11B6.2 and 13A5.2 also recognized BLG after blotting (Figure 4B), whereas chimeric human IgE anti-BLG antibody clone 8C3.3 lacked any binding against this blotted BLG. Latter observation again indicated lower affinity of clone 8C3.3, or disruption of its reciprocal epitope after denaturation. Finally, a flow cytometric study confirmed surface human FcεRI α chain expression on RBL cells (Figure 4C). Additionally, Fcε heavy chain tails of all generated chimeric human IgE anti-BLG antibodies seemed to interact with this high affinity human FcεRI as evidenced by positive flow cytometric staining (Figure 4C).

Altogether, our generated panel of non- and cross-blocking (against domains I-III) chimeric human IgE anti-BLG antibodies seemed to be of (i) high purity, and showed (ii) a proper heavy and light chain assembly into intact human IgE antibodies, while (iii) retaining their binding potential to native bovine BLG and to membrane-bound human FcεRI α chains.

### Sensitization of RBL-huFcεRI cells with the pool of six BLG-specific chimeric human Ige monoclonal antibodies and cross-linking with anti-human Ige, BLG, whey and milk powder

**Figure 5:**
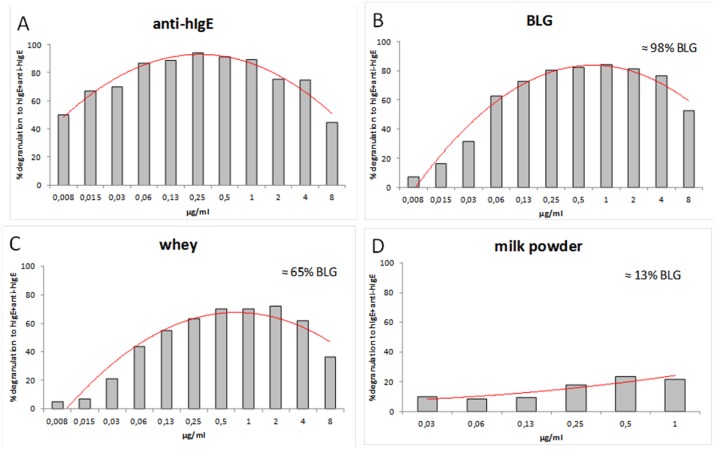
RBL-huFcεRI assay with the pool of BLG-specific chimeric human IgE monoclonal antibodies. Human FcεRI α chain expressing RBL cells were incubated with the serially-diluted pool of chimeric huIgE anti-BLG antibodies (ranging from 0.008 to 8 µg/ml of each individual huIgE; X-axis), and subsequently cross-linked with anti-huIgE antibodies (A; 5 µg/ml), BLG (B: 1 µg/ml), whey (C; (1 µg/ml), and milk powder (D; 1 µg/ml). The percentage of BLG content in each examined preparation is indicated. Results (n = 1) are representative of 1–4 independent experiments.

To investigate whether the BLG-specific chimeric IgE monoclonal antibodies were biological active, RBL-huFcεRI cells were sensitized with a serially-diluted (0.065–40 µg/ml) individual BLG-specific chimeric human IgE monoclonal antibodies, and then cross-linked with anti-human IgE. All individual BLG-specific chimeric IgE monoclonal antibodies induced degranulation with an optimum at approximately 1.0 µg/ml (data not shown). To assess the optimal concentration of our pool of six BLG-specific chimeric human IgE monoclonal antibodies (i.e., a combination of clones 5D6.1, 7D5.1, 8C3.3, 8F7.1, 11B6.2 and 13A5.2), RBL-huFcεRI cells were sensitized with serially-diluted pool of chimeric human IgE antibodies, i.e., 0.008–8 µg of each human IgE/ml for anti-human IgE, BLG and whey cross-linking, and 0.03–1 µg of each human IgE/ml for milk powder cross-linking. Cross-linking with anti-human IgE showed a maximal degranulation of 94% at a concentration of 0.25 µg of each chimeric human IgE/ml, whereas cross-linking with BLG showed a maximal degranulation of 84% at a concentration of 1.0 µg of each chimeric human IgE/ml (Figure 5). For further experiments with our pool of chimeric human IgE antibodies, a concentration of 1.0 µg of each chimeric human IgE/ml (i.e., 6 µg/ml in total) was used. To investigate whether chimeric IgE monoclonal antibodies were specific for BLG, RBL-huFcεRI cells were sensitized with BLG-specific chimeric IgE monoclonal antibodies, and subsequently cross-linked with whey (containing≈65% BLG) or milk powder (containing≈13% BLG). Cross-linking with whey showed a maximal degranulation of 70%, whereas exposure to milk powder resulted in a degranulation of only 23%, indicating that milk proteins like ALA and caseins were not involved in the degranulation and the chimeric IgE monoclonal antibodies were specific for BLG. The degranulation of the RBL-huFcεRI cells sensitized with the BLG-specific chimeric IgE monoclonal antibody pool but without cross-linking was <7% (data not shown). To further delineate the specificity of these BLG responses, degranulation from BLG-specific chimeric IgE monoclonal antibody pool-sensitized RBL-huFcεRI cells was abrogated using the cross-linker BLG (at 1.0 µg/ml), which was pre-absorbed for 15 minutes at RT with a ≈10-fold molar excess of pooled BLG-specific chimeric IgG1 monoclonal antibody counterparts of clone 5D6.1, 7D5.1, 8C3.3, 8F7.1, 11B6.2 and 13A5.2 (at 100 µg/ml in total; data not shown). In addition, the presence of therapeutic blocking anti-IgE humanized (huIgG1κ) antibody omalizumab during the sensitization of RBL-huFcεRI cells with our pool of six BLG-specific chimeric human IgE monoclonal antibodies (fixed IgE concentration of 6 µg/ml in total) dose-dependently inhibited BLG-provoked degranulation, i.e., 100% inhibition at an omalizumab-to-chimeric human IgE molar ratio of 2∶1, 76% inhibition at an omalizumab-to-chimeric human IgE molar ratio of 1∶2, and ≤10% inhibition at omalizumab-to-chimeric human IgE molar ratios of 1∶5 and 1∶15 (data not shown).

### Sensitization of RBL-huFcεRI cells with the pool of six BLG-specific chimeric human Ige monoclonal antibodies and cross-linking with whey hydrolysates

**Figure 6:**
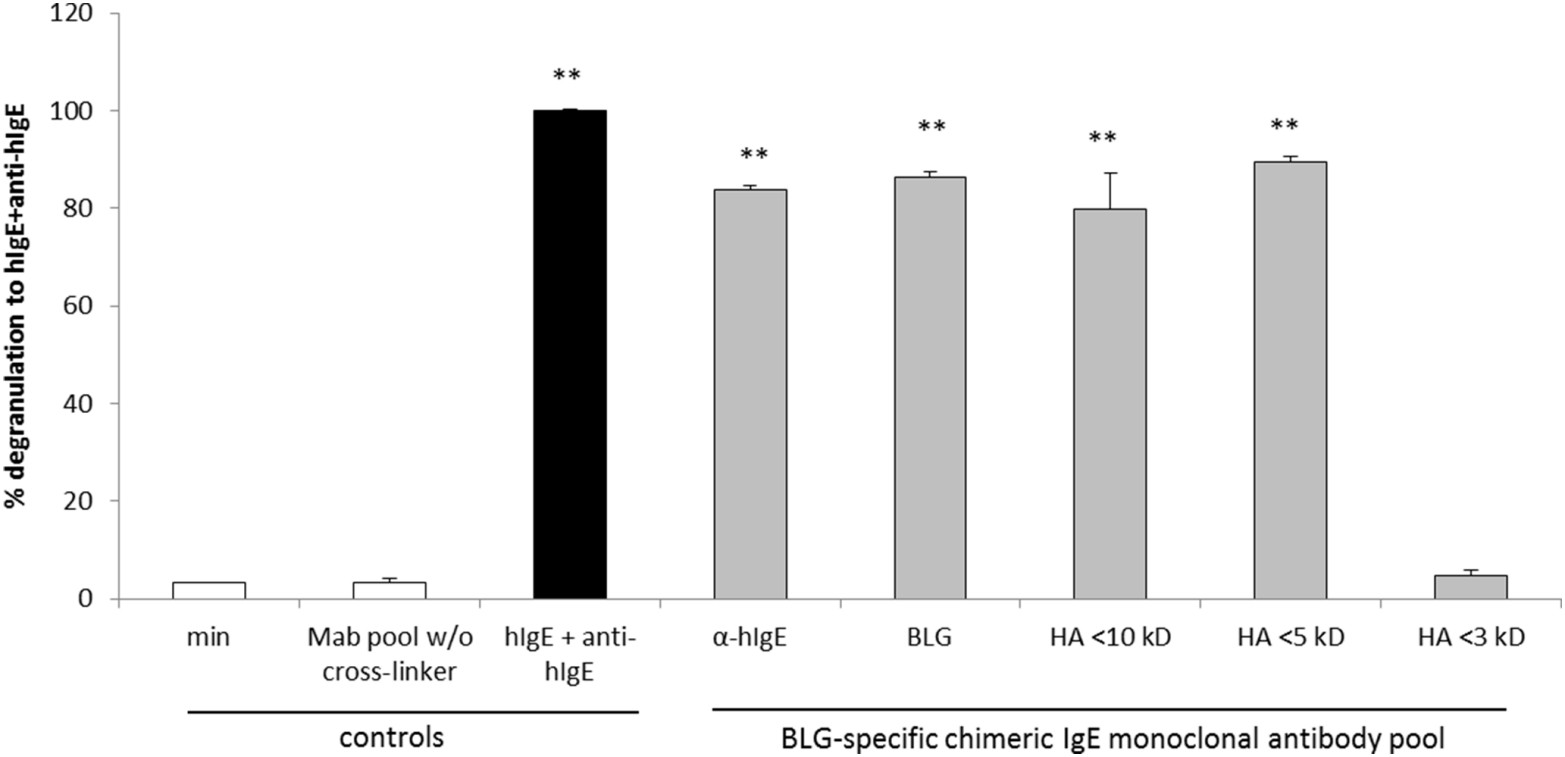
RBL-huFcεRI assay with whey hydrolysates with different peptide size patterns. Human FcεRI α chain expressing RBL cells were incubated with the pool of chimeric huIgE anti-BLG antibodies (1 µg/ml of each individual huIgE; grey bars), and subsequently cross-linked with anti-huIgE antibodies (5 µg/ml), BLG (1 µg/ml) or whey hydrolysates (1 µg/ml) with different peptide size patterns (HA<10 kDa, HA<5 kDa or HA<3 kDa). Controls: spontaneous degranulation (white bars), i.e., FcεRI α chain expressing RBL cells alone or incubated with pool of chimeric huIgE anti-BLG antibodies (Mab pool) w/o cross-linking, and maximum degranulation (black bar), i.e., FcεRI α chain expressing RBL cells treated with commercial huIgE and anti-huIgE antibodies (both at 5 µg/ml). Results are expressed as mean + SD (n = 2–6); **P<0.001 when compared to MAb pool w/o cross-linker.

To assess the sensitivity of the RBL-huFcεRI assay with the pool of six BLG-specific chimeric IgE monoclonal antibodies (i.e., a combination of clones 5D6.1, 7D5.1, 8C3.3, 8F7.1, 11B6.2 and 13A5.2), RBL-huFcεRI cells were sensitized with BLG-specific chimeric IgE monoclonal antibodies (1.0 µg of each human IgE/ml) and cross-linked with whey hydrolysates with different degrees of hydrolysis as determined by gel permeation chromatography (data not shown). The degranulation of the BLG-specific chimeric IgE monoclonal antibody pool without cross-linking was comparable to the spontaneous degranulation (min) of the RBL-huFcεRI cells alone (both 3%; Figure 6). Cross-linking the BLG-specific chimeric IgE monoclonal antibody pool with anti-human IgE antibodies and BLG showed a degranulation of 84% and 86% respectively. Cross-linking the BLG-specific chimeric IgE monoclonal antibody pool with a whey hydrolysate with peptides of <10 kDa (HA<10 kDa) and a whey hydrolysate with peptides of <5 kDa (HA<5 kDa) showed degranulation comparable to BLG (80% and 90% respectively), whereas a whey hydrolysate with peptides of <3 kDa (HA<3 kDa) showed degranulation comparable to the spontaneous degranulation (min; 5%). This showed that peptides in HA<3 kDa were not large enough to induce cross-linking of IgE or epitopes that were recognized by our chimeric human IgE antibodies were destroyed, whereas larger peptides in HA<10 kDa and HA<5 kDa were still able to induce degranulation.

### Comparison of the pool of six BLG-specific chimeric human Ige monoclonal antibodies with a serum pool of CMA patients the RBL-huFcεRI degranulation assay

**Figure 7:**
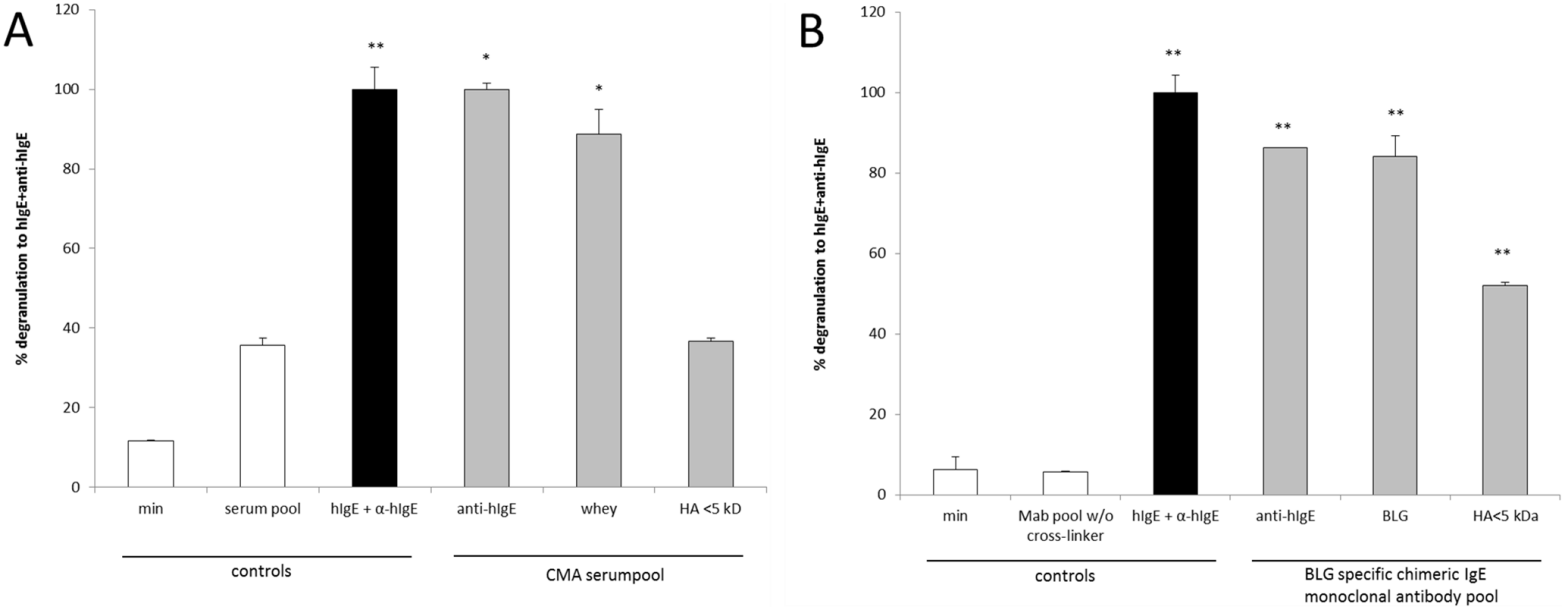
Comparison of serum versus the pool of six BLG-specific chimeric IgE monoclonal antibodies in the RBL-huFcεRI assay. Human FcεRI α chain expressing RBL cells were incubated (in two separate assays) with a pool of serum from CMA patients (A; dilution 1∶50; grey bars) or the pool of six BLG-specific chimeric IgE monoclonal antibodies (B; 1 µg/ml of each individual huIgE; grey bars), and subsequently cross-linked with anti-huIgE antibodies (5 µg/ml), whey (1 µg/ml) or BLG (1 µg/ml), and whey hydrolysate (1 µg/ml; HA<5 kDa). Controls: spontaneous degranulation (white bars), i.e., FcεRI α chain expressing RBL cells alone or incubated with pooled serum pool (A; serum pool) or chimeric huIgE anti-BLG antibodies (B; Mab pool) w/o cross-linking, and maximum degranulation (black bars), i.e., FcεRI α chain expressing RBL cells treated with commercial huIgE and anti-huIgE antibodies (both at 5 µg/ml). Results are expressed as mean + SD (n = 2–4); *P<0.05 and **P<0.001 when compared to serum pool (A) or MAb pool w/o cross-linker (B).

Direct comparison of BLG-specific chimeric IgE monoclonal antibodies with a serum pool of CMA patients was done with HA<5 kDa as a cross-linker. As shown in Figure 7, sensitization of RBL-huFcεRI cells with the CMA serum pool without cross-linking showed a relatively high degranulation (36%) compared to the spontaneous degranulation (min; 11%), whereas cross-linking with HA<5 kDa showed no additional degranulation (37%). In contrast, sensitization with our six BLG-specific chimeric IgE monoclonal antibodies (i.e., a combination of clones 5D6.1, 7D5.1, 8C3.3, 8F7.1, 11B6.2 and 13A5.2) resulted in a very low degranulation (6%) without cross-linking, whereas an additional degranulation (52%) after cross-linking with HA<5 kDa was found. This demonstrated that the use of our pool of well-defined BLG-specific chimeric IgE monoclonal antibodies increases the sensitivity of the assay.

### Epitope mapping of the six BLG-specific chimeric human IgE monoclonal antibodies and 10 sera of CMA patients

**Figure 8:**
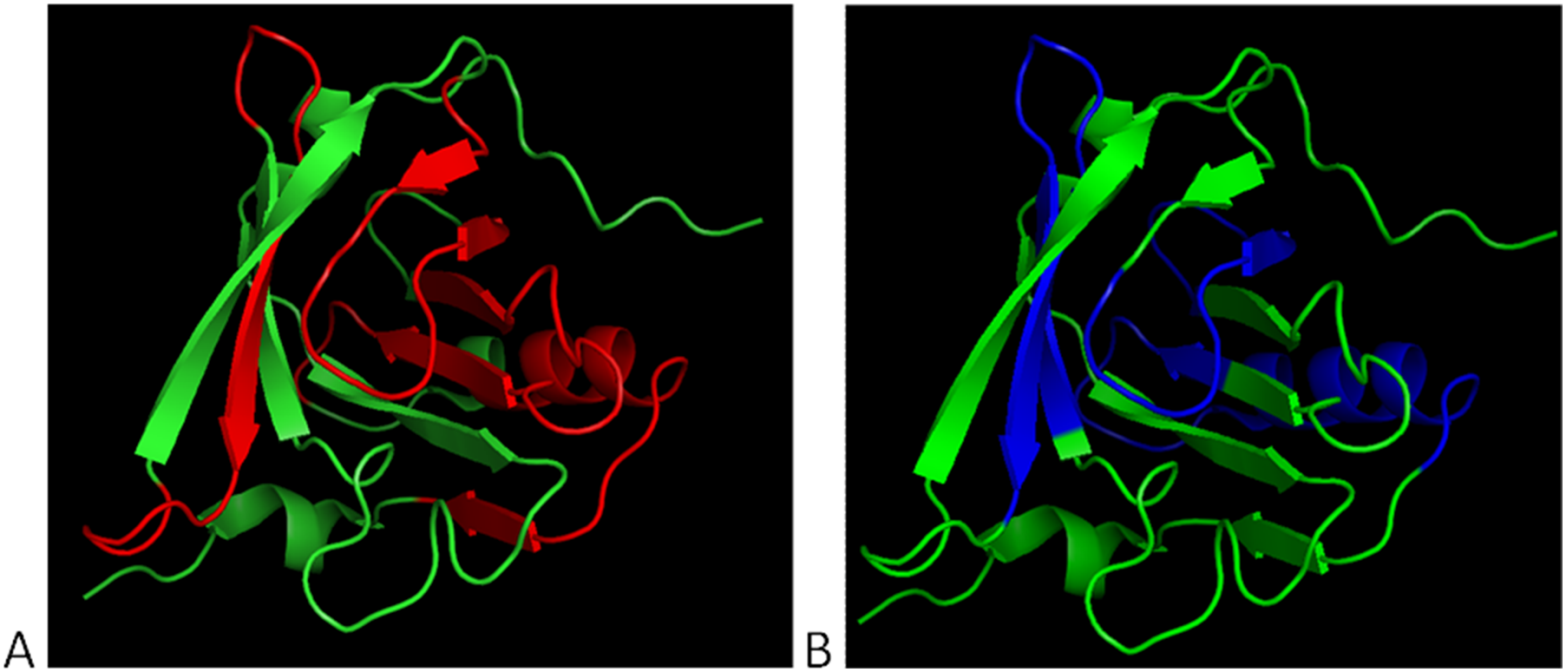
Orientation and comparison of the regions recognized by BLG-specific chimeric IgE monoclonal antibodies (A: BLG regions in red) or CMA serum (B: BLG regions in blue) in a three-dimensional ribbon structure of BLG. Pictures of the three-dimensional ribbon structure of the BLG protein created by using PyMol molecular visualization system version 1.5.

In order to assess whether the BLG-specific chimeric IgE monoclonal antibodies recognized similar regions or epitopes on BLG as IgEs from CMA allergic patients did, individual BLG-specific chimeric IgE monoclonal antibodies and 10 individual CMA sera were tested for their binding on 25 synthetic peptides spanning the BLG of the B whey protein variant and 6 peptides of the A whey protein variant. The BLG-derived peptides that were recognized by chimeric human IgEs and CMA-derived IgEs are listed in Table 3, and the orientation of these peptide sequences in a BLG three-dimensional ribbon structure is shown in Figure 8. The regions on BLG recognized by BLG-specific chimeric IgE monoclonal antibodies (Figure 8A) were very similar to the ones recognized by serum IgEs from CMA patients (Figure 8B), indicating the BLG-specific chimeric IgE monoclonal antibodies were directed to relevant allergenic regions present on BLG.

**Table 3:** BLG-derived peptides recognized by the BLG-specific chimeric human IgE monoclonal antibodies and 10 individual CMA serum samples (i.e., peptide recognition by ≥3/10 sera).

BLG-specific chimeric human IgE clone	CMA serum (peptide recognition by 3/10 sera)
Peptide: **TPEGDLEILLQK**WENDEC (AA 49–66) Clone ID: 5D6.1; 8C3.3; 8F7.1; 11B6.2	VEELKP**TPEGDLEILLQK** (AA 43–60)
Peptide: PAVFKI**DALNENKVLVLD** (AA 79–96) Clone ID: 8C3.3	**DALNENKVLVLD**TDYKKY (AA 85–102)
Peptide: LLFCMENSAEPEQSLVCQ (AA 103–120) Clone ID: 5D6.1; 8C3.3; 8F7.1; 11B6.2	
Peptide: NSAEPEQSLVCQ**CLVRTP** (AA 109–126) Clone ID: 5D6.1; 8C3.3; 8F7.1; 11B6.2	**CLVRTP**EVDDEA**LEKFDK** (AA 121–138)
Peptide: **LEKFDKALKALP**MHIRLS (AA 133–150) Clone ID: 7D5.1; 13A5.2	EVDDEA**LEKFDKALKALP** (AA 127–144)

**Identical amino acid (AA) sequences are indicated in bold, and the position on BLG is listed between brackets.**

## Discussion

Accumulating concerns about food safety have increased the attention for legislation around health claims on food products. These health claims need to be substantiated with experimental data, and for HA formulas, the elimination of potential allergic epitopes needs to be confirmed. For safety assessment of CM hydrolysates, a robust and reproducible functional assay for determination of (non)allergenicity is essential. Currently, degranulation of human FcεRI-transfected RBL-2H3 cells after sensitization with IgE from pooled serum from CMA patients and exposure to hydrolysates is being used. However, serum samples from CMA patients may contain intervening or cytotoxic substances, which make these sera less suitable for usage in this degranulation assay. In addition, limited availability and inter-lot differences (e.g., because of variable levels of allergen-specific IgE or of intervening and cytotoxic factors) of these serum pools are structural standardization issues. In the current study, we have tackled these serum-related problems by using recombinant DNA technology, and developed six BLG-specific chimeric human IgE monoclonal antibodies covering relevant allergenic epitopes of BLG, which resulted in enhanced sensitivity and specificity of the degranulation assay. Due to the recombinant nature of our BLG-specific chimeric human IgE antibodies, the sensitizing IgE pool is characterized by identicalness and *ad infinitum* availability, and hence, will improve the reproducibility of the degranulation assay.

The results of the current study unambiguously demonstrated that sensitization of FcεRI expressing RBL cells with our well-defined non-cross-blocking and cross-blocking chimeric human IgE anti-BLG antibodies - designated to recognize (arbitrary) domain I, II, and III on BLG - induced degranulation after cross-linking with BLG. Furthermore, we provided evidence that these chimeric human IgE anti-BLG antibodies bound to a number of regions on BLG alike to those that are recognized by serum IgEs from individual CMA patients (Figure 8 and Table 3), which indicated that our chimeric human IgE anti-BLG antibodies were directed against relevant ‘allergenic’ immunodominant regions on BLG. Recently, five major linear BLG epitopes/regions, covering sequences of 12–14 amino acids in length, have been identified by polyclonal serum IgEs from CMA patients [26], [27]. Interestingly, four of these five major ‘allergenic’ BLG epitopes/regions were shown to be partly overlapping epitopes/regions found with our chimeric human IgE anti-BLG antibodies, which also underscored the allergenic relevance of our generated human IgE anti-BLG antibodies. Ideally, we wanted to cover all ‘allergenic’, i.e., minor and major, epitopes on BLG [26], [27] with our pool of chimeric human IgE anti-BLG antibodies, but even when pooled serum from CMA patients is used, certain specificities will most likely be overlooked depending on the serum lots. Importantly, the *in vitro* RBL degranulation assay combined with our pool of chimeric IgEs is one of many tests, which is used for assessment of residual allergenicity of whey hydrolysates (next to peptide size distribution analysis, and residual allergen detection by ELISA and SDS-PAGE/western blotting).

Current chimeric human IgE antibodies specifically recognize BLG, which is the major allergen (≈65%) present in bovine whey. ALA is another allergen (≈25%) in bovine whey. Antibodies against ALA were also raised during our immunization with whey. These mouse anti-ALA antibodies could be used to generate chimeric human IgE anti-ALA antibodies, and subsequently implemented in the RBL degranulation assay. CM protein content consists of ≈20% whey and ≈80% casein. We first focused on whey proteins to test whey-based hydrolysates, but currently we are also developing chimeric human IgE antibodies against casein (i.e., αS1, αS2, β, and κ casein) to investigate (non)allergenicity of casein-based hydrolysates in the RBL degranulation assay.

Theoretically, cross-linking of FcεRI/IgE complexes by monomeric allergens on mast cells or basophils is achieved by at least two IgE antibodies whose binding sites on an allergen are not overlapping, although homodimeric (including BLG) or even oligomeric allergens have been described to enhance allergenicity [28]. Therefore, we primarily aimed to generate a set of non-cross-blocking anti-BLG antibodies. However, since we did not measure affinity or dissociation constants of our chimeric human IgE anti-BLG antibodies, and a mixture of our non- cross-blocking and cross-blocking chimeric human IgEs could possibly mimic polyclonal anti-BLG IgEs in CMA more closely, we decided to use the six chimeric human IgE anti-BLG antibodies simultaneously for sensitization of FcεRI expressing RBL cells. In principle, cross-blocking antibodies do not necessarily bind to identical epitopes, which was confirmed by the peptide ELISA, but obviously, various combinations of our chimeric human IgE anti-BLG antibodies remain to be investigated in the RBL degranulation assay for optimizing purposes (e.g., enhancement of sensitivity and/or degranulation responses). Irrespective the outcome of this optimizing step, the current combination of all six chimeric human IgE anti-BLG antibodies demonstrated excellent and very sensitive – far more than obtained with serum IgEs from CMA patients - degranulation responses from RBL cells using bovine whey, BLG and whey hydrolysates (5–10 kDa) as cross-linkers. Especially, avoidance of potentially inhibitory and cytotoxic factors [13], [18], [19], [29], which are likely to be present in serum samples from allergic patients, seemed to be an enormous advantage of using our ‘clean’ pool of chimeric human IgE anti-BLG antibodies in the RBL degranulation assay.

As mentioned above, dimeric allergens are suggested to increase allergenicity [28], because it would need only two identical IgE antibodies, instead of two or more IgE antibodies against dissimilar epitopes, for cross-linking and subsequent mast cell or basophilic cell activation. Despite the presence of minute amounts of dimeric BLG in our whey preparation (data not shown), we observed no or insignificant degranulation from RBL cells that were sensitized with only one of our chimeric human IgE anti-BLG antibodies (data not shown). However, sensitization of RBL cells with individual chimeric human IgE anti-BLG antibody could possibly be used for quality testing of whey hydrolysates, i.e., aggregate formation.

As shown in previous experiments in which whey protein degradation was followed during the hydrolysis process, a concurrent degradation of BLG and ALA was shown [23]. This study also showed that the capacity to cause IgE-mediated mast cell activation is size dependent. For IgE cross-linking, it has been concluded from several studies that the distance between two FcεRI molecules is within 8–24 nm, corresponding to approximately 30–100 amino acids (≈3.3–11 kDa) [2]. Peptides below this size are unable to cross-link IgE on mast cells. To test this in our RBL-huFcεRI degranulation assay with the pool of six BLG-specific chimeric human IgE monoclonal antibodies, whey hydrolysates with a different peptide size pattern were analysed. It was clearly shown that the hydrolysate with peptide sizes smaller than 3 kDa did not elicit any degranulation, whereas the hydrolysates with peptides smaller than 10 kDa, and also smaller than 5 kDa, did show substantial degranulation in our assay. Therefore, this degranulation assay in combination with our pool of BLG-specific chimeric human IgE monoclonal antibodies, which may serve as a model for the effector phase of the allergic response, could be a highly relevant and sensitive *in vitro* model to assess the safety of and to improve hydrolysed formula for infants diagnosed with CMA.
